# Prolonged Mechanical Ventilation At Home Versus Long Term Care: Caregiver Characteristics And Strain

**DOI:** 10.1093/geroni/igab046.2941

**Published:** 2021-12-17

**Authors:** Esther-Lee Marcus, Jeremy Jacobs, Jochanan Stessman

**Affiliations:** 1 Herzog Medical Center, Herzog Medical Center, Yerushalayim, Israel; 2 Hebrew University of Jerusalem, Jerusalem, Yerushalayim, Israel; 3 Hadassah Hebrew University Medical Center, Clalit Health Services, Yerushalayim, Israel

## Abstract

Although the number of Prolonged Mechanical Ventilation (PMV) patients and their informal caregivers (CGs) is rising both at Home or Long Term Care (LTC), little is known concerning CG characteristics or strain. We enrolled 120 patients and 106 informal CGs: 34/46 and 72CGs/74 PMV patients from Home Hospital and LTC respectively. CGs were married (82%), female (60.4%), mean age 59 ±14 years; spouses (29%) or children (40%) of the PMV patient. The 13-item Modified Caregiver Strain Index (MCSI) (Maximum severity=26) was 13.6± 6.5, similar at Home vs. LTC (14.3±7.5 vs. 13.3±6.0, p=0.9). Most frequent complaints were distress concerning patient’s changes (93%) or upsetting behaviours (82%), feeling overwhelmed (82%), sleep disturbance (69%) and emotional adjustments (67%). Home CGs reported significantly more physical and financial burden, confinement, and need for work adjustment, while LTC CGs reported greater emotional disturbance and upsetting patient behaviours. Hierarchical clustering identified three clusters of CG strain: burden (physical/time/financial), emotional (upsetting adjustment/ behaviours/overwhelmed) and disturbance (work/plans/confinement). Emotional strain was most frequent, irrespective of site of care; however CGs at Home vs. LTC experienced significantly higher burden and disturbance vs. higher emotional strain respectively. In multivariate models, after adjusting for numerous patient and CG variables, increasing CG strain was consistently associated with rising patient symptomatology. This relationship was pronounced among CGs of Home PMV patients, with a significant interaction variable of Home*Patient symptomatology. Our findings identify specific patterns of strain among caregivers of PMV patients whether at home or LTC, and highlight the importance of addressing their unique needs.

